# School-based epidemiology study of myopia in Tianjin, China

**DOI:** 10.1007/s10792-020-01400-w

**Published:** 2020-05-29

**Authors:** Jiaxing Wang, Ying Li, Zhenyang Zhao, Nan Wei, Xiaoli Qi, Gang Ding, Xue Li, Jing Li, Linlin Song, Ying Zhang, Richard Hyun Yi, Yuxian Ning, Xiaoyu Zeng, Ning Hua, Xuehan Qian

**Affiliations:** 1 Department of Ophthalmology, Emory University, Atlanta, GA USA; 2 Department of Ophthalmology and Visual Sciences, University of Texas Medical Branch, Galveston, TX USA; 3 Department of Strabismus and Pediatric Ophthalmology, Tianjin Medical University Eye Hospital, Tianjin, 200284 China; 4 Medical College of Georgia, University of Georgia Health Sciences, Athens, GA USA

**Keywords:** Myopia, Epidemiology, Children, Refractive error, Visual acuity, Photoscreen

## Abstract

**Purpose:**

To study the epidemiology of myopia in school-aged children in Tianjin and the relationship between visual acuity-based screening and refraction-based screening.

**Method:**

This school-based prospective cohort study was performed on children from 42 elementary schools and 17 middle schools in Tianjin, China. Totally 14,551 children, ages ranging from 5 to 16 years, were included in this study. Uncorrected visual acuity (UCVA) was determined by logarithmic tumbling E chart. Non-cycloplegic photorefraction was examined by the Spot (v2.1.4) photoscreener. The relationship between the UCVA and refractive error was investigated for different age groups.

**Results:**

The overall prevalence of myopia at this school based screen is 78.2%, ranged from 10% at age of 5 to 95% at age of 16. The most dramatic increase in prevalence is from age of 6 (14.8%) to age of 7 (38.5%). The overall prevalence of high myopia is 2.5%. UCVA is found corresponding to spherical equivalent refraction (SER) in a manner of normal distribution and is significantly affected by age. When using UCVA to estimate the prevalence of myopia, the overall sensitivity and specificity are 0.824 and 0.820, respectively. Age-dependent optimal cutoff points and 95% confident intervals of such estimation are reported.

**Conclusions:**

Myopia is heavily affecting school-aged children in Tianjin, China. The refraction screening is preferable for myopia screening, whereas the UCVA screening results need to be interpreted in an age-dependent manner for myopia estimation.

**Electronic supplementary material:**

The online version of this article (10.1007/s10792-020-01400-w) contains supplementary material, which is available to authorized users.

## Background

With rapidly increasing prevalence in the past few decades, myopia is becoming a major health issue around the world and is heavily affecting East Asia [1]. Epidemiological studies of myopia are usually performed with population-based refraction screening as well as vision screening. Uncorrected visual acuity (UCVA) was used to estimate the prevalence of myopia in many countries including China [2–4]. It has the advantage of fast screening and low cost, while the disadvantages include subjective test as well as relative low specificity, since an decreased UCVA can be affected by conditions other than myopia such as hyperopia, astigmatism or amblyopia.

Regarded as a more objective measurement, refractive testing has been employed in emerging studies to identify myopia within a population. However, problem appears in large-scale screening since cycloplegic refraction, the consented gold standard, is time-consuming and requires trained professionals. This issue has been addressed with the development of photoscreeners, which offer the balance of both accuracy and speed, making them ideal for large population refraction screening. It has been supported by multiple studies, including ours, showing similar outcomes after comparing photoscreeners with cycloplegic refraction exams [5–9]. The Spot photoscreener we used in this study is a newly developed portable handheld infrared photoscreener (Welch Allyn, Skaneateles Falls, NY). Red reflex images can be acquired from subjects by simply asking them to look at the device, while non-cycloplegic refractive status, pupil size and gaze deviation are automatically recorded. It is reported to have an overall high sensitivity (91.7%) and specificity (82.6%) in detecting amblyogenic risk factors in children [10], which makes it an ideal screener for myopia.

Tianjin is one of the 4 municipalities in northern China with a total population of 15,621,200 as of 2016 estimation. It follows the existing Chinese education system which consists of one-year pre-elementary school starting from age of 5, 6 years of elementary school starting from age of 6, 3 years of junior middle school and 3 years of senior middle school before college. In this study, we performed a school-based screening for 14,551 school-aged children in Tianjin on both the UCVA and refractive error, aimed to investigate the prevalence of myopia as well as the relationship between UCVA and refractive error in this population.

## Methods

### Subjects

This school-based prospective cohort study was approved by the Ethics Board of Tianjin Medical University Eye Hospital. Informed written consent was obtained prior to the start of the study from parents of all participants according to the Declaration of Helsinki. All questions and concerns were addressed before the consent forms were signed. The screening was performed on children from 42 elementary schools and 17 middle schools in Tianjin, China, from February to May in 2018. Children from ages of 5 to 16 years were recruited.

## Visual acuity test and refraction screening

The screen date is informed to the school at least one week ahead to let the subjects be prepared. The subjects are required not to wear spectacle glasses but not contact lens on screen day. The students with current corneal refractive therapy (Ortho-K) were also asked not to wear the Ortho-K the night before the screen date and wear spectacle glasses the screen date instead. The subjects are asked to remove the spectacle glasses for the uncorrected visual acuity test and the refraction test by Spot photoscreener.

Uncorrected visual acuity (UCVA) was tested using logarithmic tumbling E chart and recorded as the smallest size that the subject can identify in all four directions. The test was conducted monocularly by trained pediatric ophthalmology residents under room light during the daytime, starting with the right eye (OD) while the left eye (OS) was occluded with a non-contact black spoon-shaped eye occluder. The child could use fingers or arms to point the direction of the opening of the “E” letter from a distance of 5 m. When the OD test is finished, the OS was tested after a 5–10 s of break time. The break was given to each child for the OS to recover from the previous occlusion.

Non-cycloplegic refractive error was tested using the Spot photoscreener. The measurement range of the Spot screener was limited to ± 7.50 D. If the refraction was out of range, ± 8.00D was recorded for further analysis. The spherical equivalent refraction (SER) was collected to determine the prevalence of myopia (SER ≤ − 0.5D), mild myopia (− 2D < SER ≤ − 0.5D), moderate myopia (− 6D < SER ≤ − 2D), high myopia (SER ≤ − 6.0D) and anisometropia (interocular difference in SER ≥ 1D). Testing was conducted by trained staff who obtain results from each child in three trials. During the test, the examiner asks the subject to look at the device binocularly from a one-meter distance. Red reflex images are acquired from subject, and non-cycloplegic refractive status, pupil size and gaze deviation are automatically recorded. Since both eyes are examined simultaneously, data acquisition is finished within 2 s [11, 12]. All screened subjects from this study were successfully tested. The software algorithm would flag a referral for complete eye examination if significant refractive error, anisometropia or strabismus were detected. Referral criteria of Spot (Software v2.1.4) are listed in Supplementary Table 1. Full ophthalmic examinations were performed on referred children by board-certified ophthalmologists. Children with optical deprivation, cataract, ptosis, glaucoma, retinal diseases or ocular trauma were excluded from this study.

## Data analysis and statistics

For the age distribution histogram, the prevalence of myopia and the comparison between right eye (OD) and left eye (OS) were analyzed for each child. For all other analysis, the individual eye was considered as an observation. The UCVA was recorded in a decimal scale and converted to logMAR for analysis [13, 14]. The relationship between UCVA and SER was analyzed. The sensitivity and specificity of detecting myopia by UCVA were calculated, and the overall effectiveness was analyzed by receiver operating characteristic (ROC) curves. The role of age was specifically considered for all of the analysis. Data are presented as the mean ± standard deviation of the mean (SEM). The analyses and figures were performed using the software R [15] version 2.5.1, and RStudio [16]. Statistical analysis was applied using the software R version 2.5.1. Student's t test, Chi-square test and one-way ANOVA test were used when appropriate, as shown with the results.

## Results

### Descriptive data and the prevalence of Myopia

**Fig. 1:**
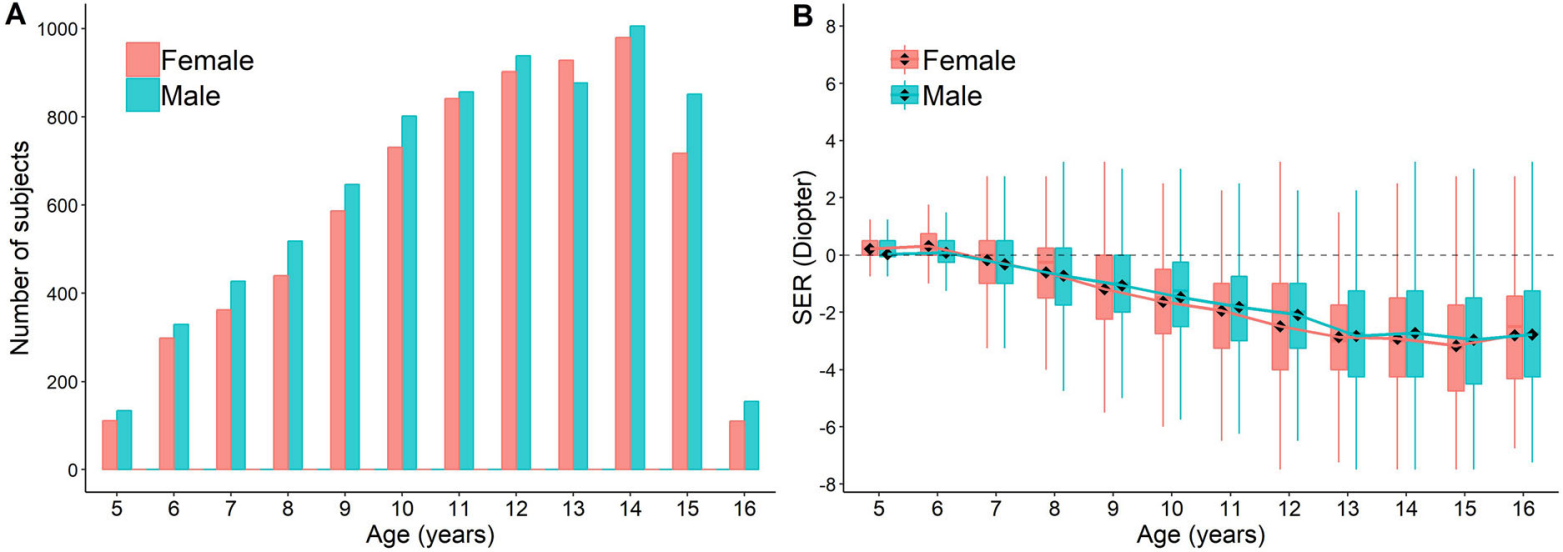
Data Distribution. The distribution of the number of subjects (**a**) and SER (**b**) across ages. Boxplots show mean value (round dot), median (horizontal line), 25^th^ and 75th percentile, maximum and minimum values for each group. The mean values are connected to show the trend of SER change with development. No statistical significance is found in any of the age between genders (Student's t test). See statistics for comparison between each age group in Supplementary Table 5

A total of 14,551 children (29,102 eyes) have been included in this study, with age ranging from 5 to 16 (mean = 11.2 ± 2.8) years (Supplementary Table 2), consisting of 7,542 (51.8%) males and 7,009 (48.2%) females. Age distribution is shown in Fig. 1a. No statistical significance is found in any of the age between genders (Student's t test). The refractive errors analyzed by age (Fig. 1b) reveal that the myopic process in this population starts from 6 to 7 years of age (0 D) and progresses till 12 years old (− 2 D). This time period corresponds to the elementary school, from the first grade to the sixth grade. The progression of refraction change slows down from age of 13 and afterward (See statistics in Supplementary Table 3). The data suggest that initiation and progression of myopia occur most frequently during elementary school age.

**Fig. 2:**
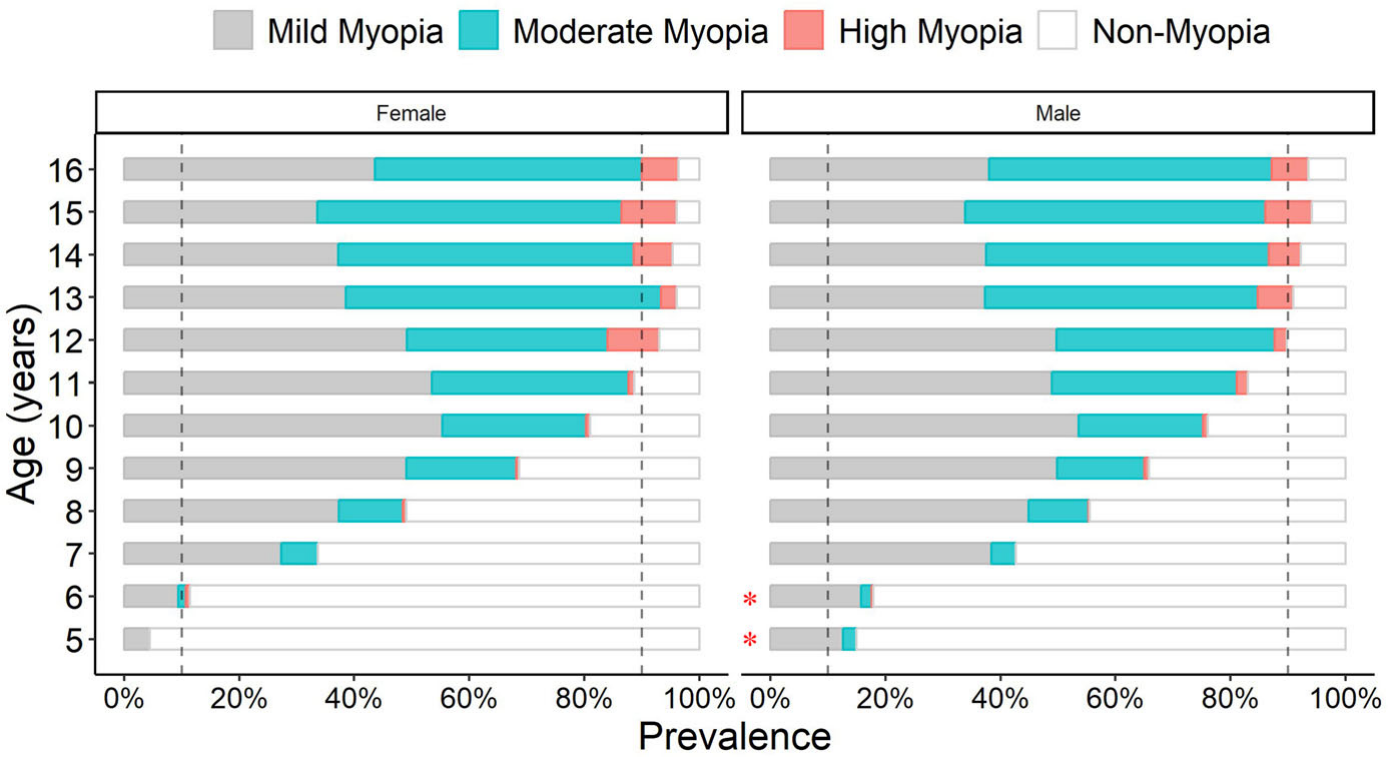
The prevalence of myopia in different age groups. Two vertical dashed lines indicate the prevalence of 10% and 90%, respectively. Mild myopia: − 2D < SER ≤ − 0.5D; moderate myopia: − 6D < SER ≤ − 2D; high myopia: SER ≤ − 6D; non-myopia: SER > − 0.5D. *p < 0.05 (Chi-square test, between genders)

The overall prevalence of myopia at this school based screen is 78.2%. The age-stratified prevalence is 10.2% for age of 5, 14.8% for age of 6, 38.5% for age of 7, 52.6% for age of 8, 67.2% for age of 9, 78.4% for age of 10, 85.8% for age of 11, 91.4% for age of 12, 93.5% for age of 13, 93.8% for age of 14, 95.0% for age of 15 and 94.7% for age of 16. The most dramatic increase in prevalence is at 7 years old, where the prevalence almost tripled when comparing with age of 6. The prevalence increases gradually and reaches 90% by 12 years old. There is a significant difference in the prevalence of myopia between males and females at both ages of 5 and 6 (Chi-square test, *p* < 0.05, Fig. 2). No significant difference is found between genders after 7 years of age. In contrast with myopia, most dramatic increase in high myopia is at age of 12 (5.5%), where the prevalence also tripled when comparing with age of 11 (1.5%), and continue rising afterward. Detailed data are available in Supplementary Table 2.

## Relationship between the UCVA and SER

**Fig. 3:**
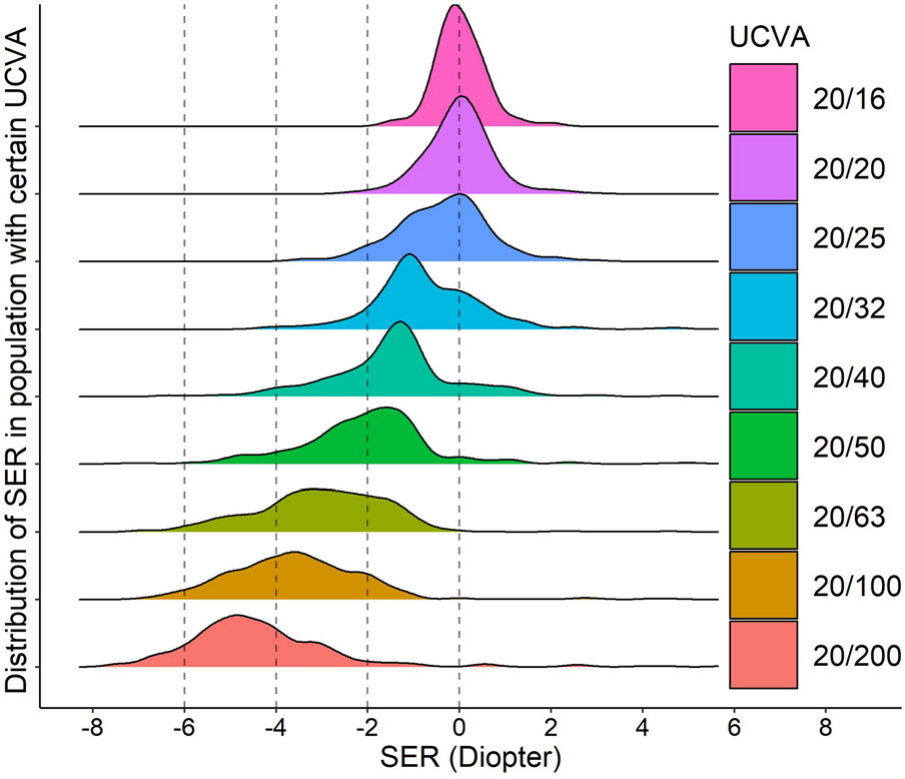
The relationship between SER and various levels of UCVA. For each level of UCVA, the colored area represents the distribution of the number of eyes for a given SER. It shows that each level of UCVA is corresponded with a certain range of SER in a manner of normal distribution. With the decrease in UCVA, the density curves shift toward more myopic and bigger variance range of SER

**Fig. 4:**
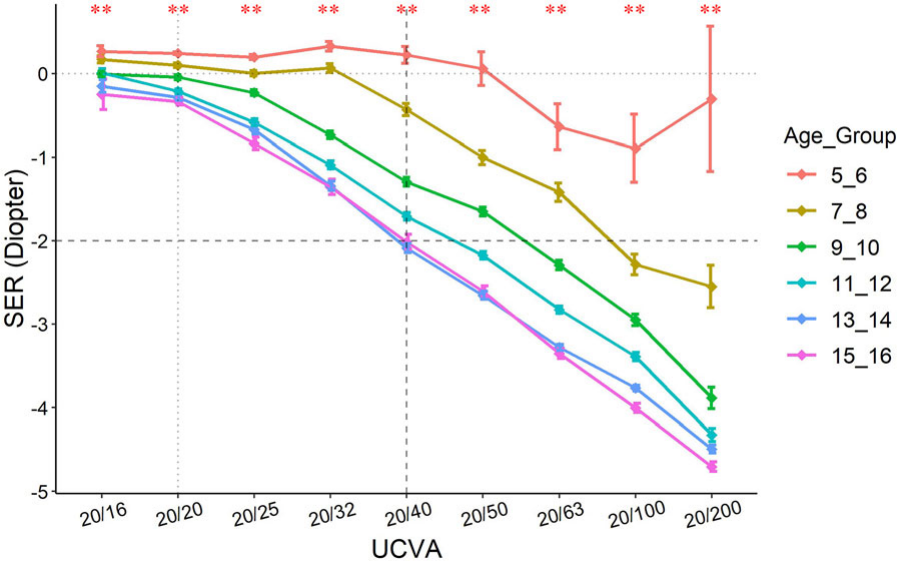
Relationship between SER and UCVA in different age groups**.** The subjects were grouped by every 2 years of age. SER of − 2D and UCVA of 20/40 were outlined by dashed lines so that their trend by age can be appreciated. **p < 0.01 (One-way ANOVA, across age groups)

When investigating the correlation of these two parameters we found that among the eyes of the similar UCVA, the SER readings follow a normal distribution (Fig. 3). The UCVA of 20/25 to 20/15 corresponds with a range of SER centered at 0D. With the decrease in UCVA, the bell curves shift left with lower and wider shapes, indicating bigger variance and myopic shift of the SER (Fig. 3). A negative shift of SER correlates with worse UCVA implying myopia as the main cause of vision impairment among the studied subjects. Further analysis showed that the SER distribution at different levels UCVA is neither gender nor laterality dependent (Supplementary Fig. 1), but strongly age dependent (Fig. 4). When grouping subjects by every 2 years of age as an interval, we found that SER more closely correlated to UCVA in older children than younger ones. With the same UCVA, children from different ages may have various SER (Fig. 4, vertical dashed line) and vice versa (Fig. 4, horizontal dashed line).

Within each age group, given the same UCVA, the SER also follows a normal distribution (Supplementary Fig. 2). A 95% confidence interval (CI) of each SER distribution is listed based on age and UCVA (Supplementary Table 4). Those with SER out of the 95% CI should be considered as a “red flag” and would substantially benefit from additional ophthalmologic assessment.

## The role of UCVA in predicting the prevalence of myopia

**Fig. 5:**
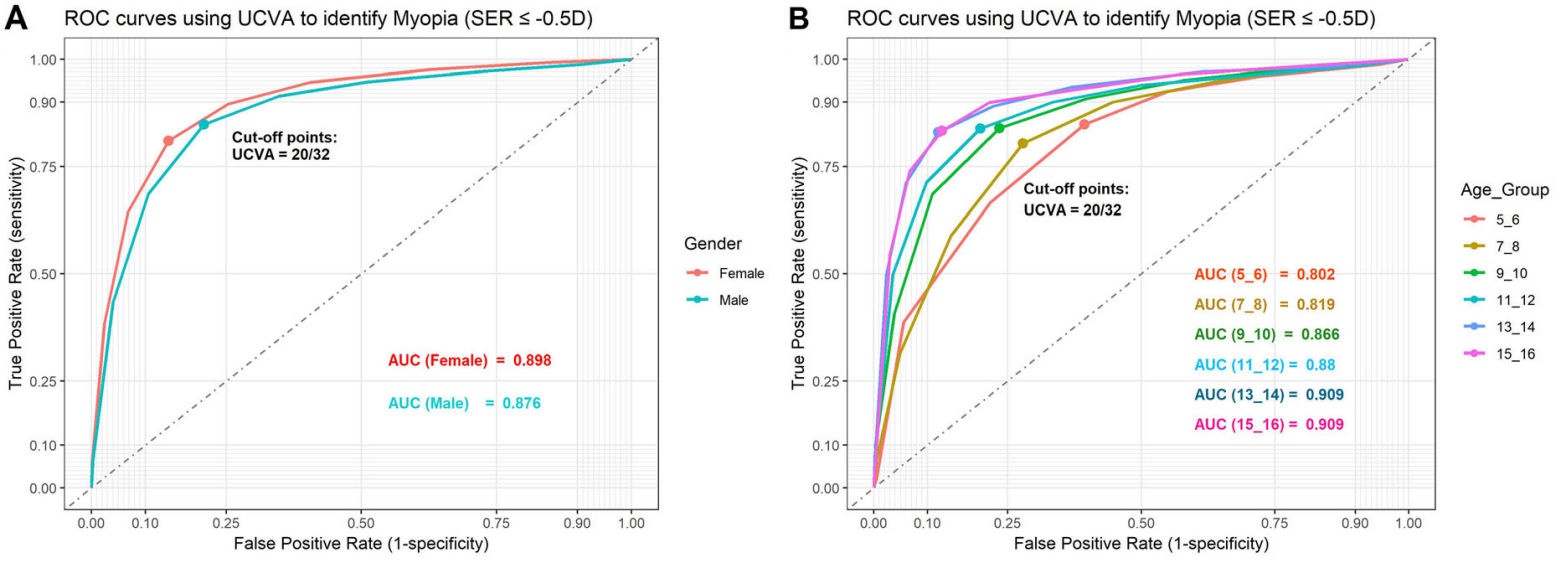
ROC plots**.** ROC plots using the UCVA to estimate the prevalence of myopia, grouped by gender (**a**) and by age (**b**). ROC: receiver operating characteristic. AUC: area under the curve

The UCVA has been used to estimate the prevalence of myopia in many countries [2, 3], and a linear relationship between UCVA and myopia has been reported [3]. Leone JF et al. showed that the prevalence of UCVA may provide a reasonably accurate estimate of the prevalence of myopia for adolescent children in Australia [17]. In this study, we generated ROC plots for predicting the SER using UCVA based on genders and ages (Fig. 5). With myopia defined as SER ≤ − 0.5D, we found that the overall AUC was 0.898 and 0.876 for females and males, respectively (Fig. 5a). The optimal cutoff points were at UCVA = 20/32, similar to previous studies [18–20], where the sensitivity and specificity are 0.824 and 0.820, respectively (Fig. 5b).

In this prediction model, age once again plays a role (Fig. 5b). Our data show that the prediction of myopia with UCVA works better in older children than their younger counterparts, indicating the involvement of visual development or cognitive development. After testing the sensitivity and specificity of different UCVA cutoff points at different ages, we listed age-adjusted optimal cutoff points of UCVA for detecting myopia (Supplementary Table 2).

## Anisometropia

**Fig. 6:**
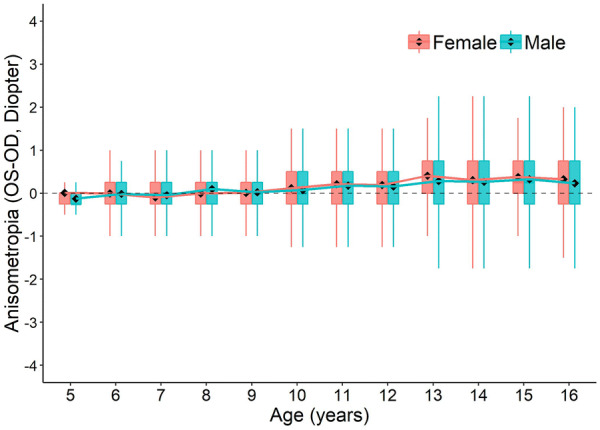
The distribution of anisometropia (SER_OS _− SER_OD_) across ages**.** Boxplots show mean value (round dot), median (horizontal line), 25th and 75th percentile, maximum and minimum values for each group. The mean values are connected to show the trend of anisometropia change with development. No statistical significance is found in any of the age between genders (Student's t test). See statistics for comparison between each age group in Supplementary Table 5

Anisometropia (ΔSER ≥ 1D) is represented by the differences between the SER in OD and OS. The average anisometropia before 10 years old is stable at 0 D, with minimal difference in SER from each eye. The prevalence of anisometropia starts to increase at the age of 11, with the OD becoming relatively more myopic than the OS (SER_OD_-SER_OS_ < 0) in both genders at all ages (Fig. 6, see statistics in Supplementary Table 5).

## Discussion

### The prevalence of myopia in Tianjin

Controversy exists in the definition of myopia. A commonly used criterion for myopia is either eye with a refraction error ≤  − 0.50D [27, 28]. Due to the large number of affected populations, any small changes in the refraction error threshold will have huge impact on estimating the prevalence of myopia and its associated risk factors [29]. In this study, we used the most widely used cutoff of ≤  − 0.50D in SER to define myopia. Due to the employment of non-cycloplegic measurements, the myopic component tends to be over-estimated in young children.

The overall prevalence of myopia in this Tianjin school-aged population is 78.2%. The progression starts to increase dramatically at 7 years old (second grade) and reaches 90% at the age of 13 (eighth grade) when the increasing slows down (Figs. 1b, 2). This period covers the entire elementary school period, suggesting a strong correlation between myopia development and intense schooling at primary school [18, 30].

## Photoscreening and corrections for photoscreener results

The Spot photoscreener we used in this study has been shown to have good consistency with cycloplegic refraction test in our previous study [5, 31, 32]. It is also reported to have an overall high sensitivity (91.7%) and specificity (82.6%) in detecting amblyogenic risk factors in children [10], suggesting its good test accuracy. This evidence proved it a reliable screener for myopia. However, given the fact that the data from non-cycloplegic photoscreeners tend to over-estimate the prevalence of myopia [18, 33, 34], the actual prevalence may be lower than reported. This has been elaborated in our previous study that the SER results from Spot photoscreener overall represent a myopic shift, with an average of − 0.17D ~ − 0.49D [9]. Based on this, we re-calculated the prevalence of myopia and high myopia in this population by modifying the SER by + 0.25D. The modified prevalence is 75.6% for myopia and 2.5% for high myopia for in this population. Theoretically, this modified prevalence closely represents the cycloplegic results. The overall over-estimation of the prevalence is 2.7% (78.2% compared with 75.6%) for myopia and 1% (2.5% compared with 2.5%) for high myopia. Such myopic shift measurement is not only seen in the Spot photoscreener, but also in other screeners such as PlusOptix A09 [35, 36] and plusoptiX A12C [37]. This increased myopic measurement could be explained by the pupil dilation or the accommodation induced by the 1 m working distance of screener [5]. It is also the reason of the relatively high referral rate for amblyopia risk factors from the photoscreeners [10, 11].

Some advantages of the refraction screen using photoscreeners are: (1) The test is quick and suitable for large populations; (2) minimal cooperation required by the subject, which makes it suitable for children with cognition disorders [12]; (3) less professional dependent; (4) electronic upload of the results provides convenience for later analysis and follow-ups; and (5) results are applicable to other conditions such as hyperopia and astigmatism. It must be pointed out that the result from photoscreeners is non-cycloplegic refraction. Although it was reported by numerous studies that the results are very close to the cycloplegic refraction [5, 9], it is not used as gold standard for diagnosing refractive error. However, this does not undermine the screening value of the photoscreeners for the reasons stated above. It is believed that with the development and improvement of the device, the results of this rapid screening will be more accurate, or closer to the results from the gold standard, which make its role in refractive screening more prominent.

## Interpretation of the UCVA based screening in an age-dependent manner

The UCVA is used to estimate the prevalence of myopia in many countries and regions, especially the underdeveloped places [2, 3]. It is critical to have an accurate interpretation of the UCVA-based myopia estimation. It has been reported that the prevalence of uncorrected VA may provide a reasonably accurate estimate of the prevalence of myopia, where a VA cutoff of 6/9.5 or less detects myopic refractive error reliably in an adolescent population [17, 38]. In this Chinese population, we also see good estimation of myopia from UCVA test with the optimal cutoff points at UCVA = 20/32, similar to other reports [17–20] (Fig. 5, Supplementary Table 2). Moreover, we found such estimation is strongly age dependent (Figs. 4, 5b), suggesting that the screening criteria should also be age dependent.

In this study, we further provided a 95% CI of SER in its respective UCVA for different ages (Supplementary Table 2). With this confidence interval, one may be able to further screen the children who may have abnormal visual-refractive development (out of the 95%CI), with only the UCVA and age. Such children are considered a higher risk of having an ocular condition beyond refractive error, which is a strong indication of ophthalmic referral.

## Anisometropia

The longitudinal increased prevalence and severity of anisometropia with increasing age we observed in this study (Fig. 6) has also been reported by many other studies, in both children [21–23] and adults [24–26]. These results suggest that anisometropia develops slowly later in childhood, with the right eye developing slightly faster than the left eye. The reason is unclear. The right eye progressed in myopia slightly faster than the left eye (Fig. 6), where it can also be seen from Supplementary Fig. 1b, with the right eye, is more myopic than the left eye for all levels of UCVA, although without statistical significance. The reasons of these findings are unclear. Studies have shown that the dominant eye may have a greater degree of myopia than the non-dominant eye in subjects with anisometric myopia [39, 40]. One other possible assumption is that the right-hand dominance is somehow contributing the more myopic in the right eye. However, currently, no significant association has been found between hand dominance and ocular dominance [39, 41].

## Limitations

Firstly, since this is a non-cycloplegic photoscreening study, the prevalence of myopia and high myopia may be over-estimated. This limitation may not affect future longitudinal studies that monitor the progression of refractive error for the same population using the same method. Secondly, part of our subjects with undetected ocular diseases may have not been appropriately excluded from this study due to insufficient information or loss of follow-up. Given the large sample size of this study, this limitation should have very little impact on the conclusion. Last, the absence of risk factors evaluation in association with myopia also adds a limitation to this study.

## Electronic supplementary material

Below is the link to the electronic supplementary material.

Supplementary file1 (JPG 416 kb)

Supplementary file2 (JPG 1599 kb)

Supplementary file3 (DOCX 30 kb)
