# Supplementary figures and images for: School-based epidemiology study of myopia in Tianjin, China
Source: Int Ophthalmol. 2020 May 29;40(9):2213–22. doi: 10.1007/s10792-020-01400-w (PMC7481173; doi:10.1007/s10792-020-01400-w)

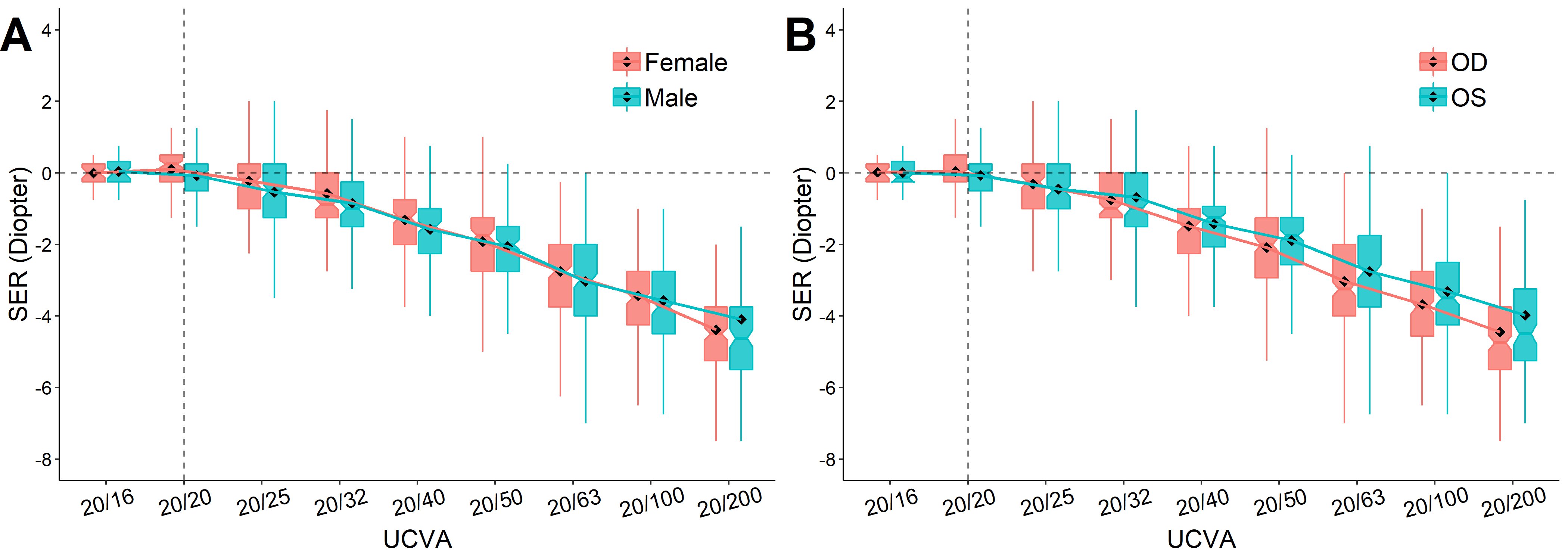

Supplement: Supplementary file 1 — Supplementary file1 (JPG 416 kb) [file 10792_2020_1400_MOESM1_ESM.jpg]

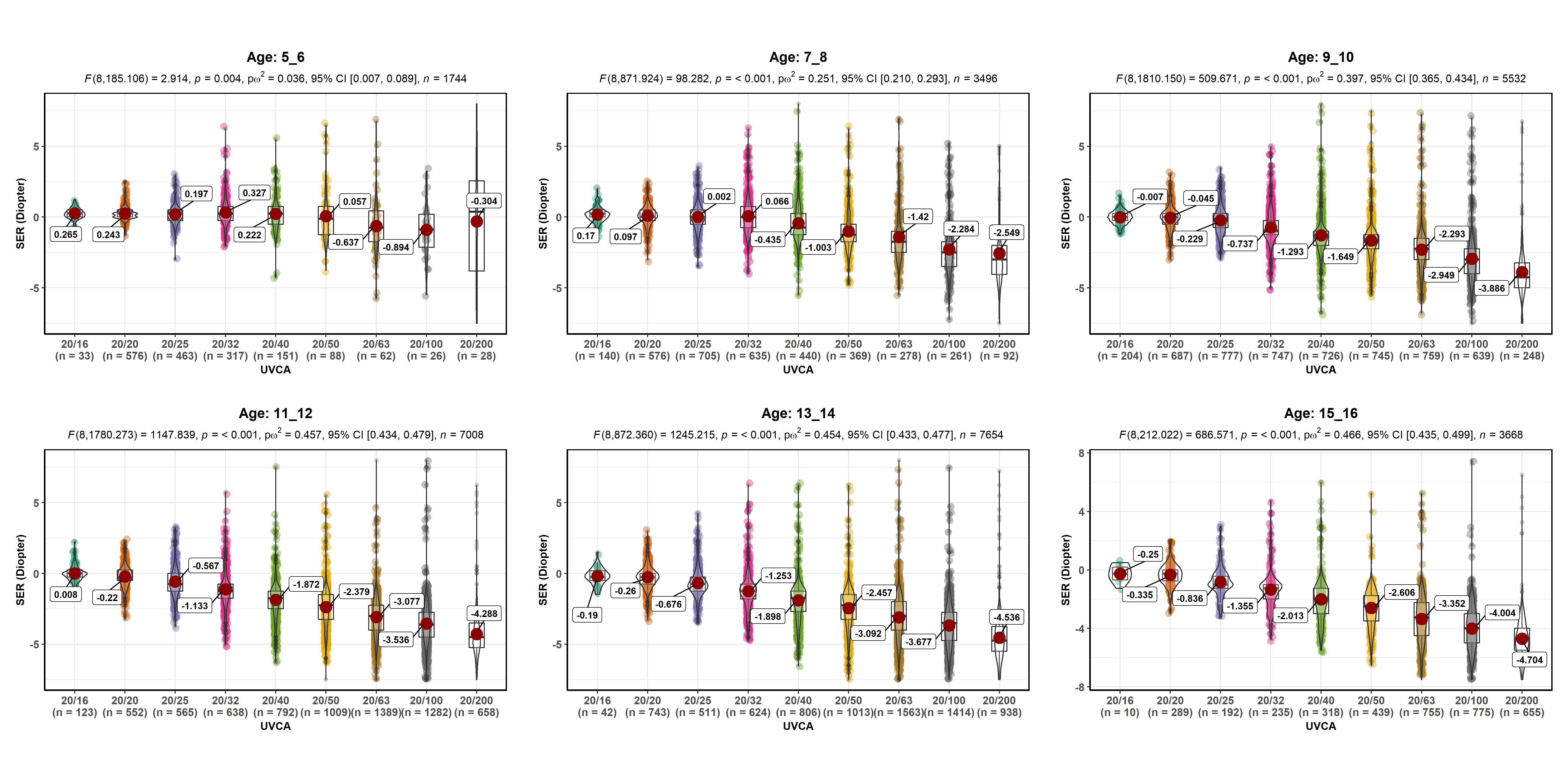

Supplement: Supplementary file 2 — Supplementary file2 (JPG 1599 kb) [file 10792_2020_1400_MOESM2_ESM.jpg]
