# Supplementary material for: School-based epidemiology study of myopia in Tianjin, China
Source: Int Ophthalmol. 2020 May 29;40(9):2213–22. doi: 10.1007/s10792-020-01400-w (PMC7481173; doi:10.1007/s10792-020-01400-w)
Supplement: Supplementary file 3 — Supplementary file3 (DOCX 30 kb) [file 10792_2020_1400_MOESM3_ESM.docx]

**Supplementary Tables:**

**Supplementary Table 1.** Referral criteria of Spot, software v2.1.4.

| Age  (Months) | Hyperopia  (D) | Myopia  (D) | Astigmatism  (D) | Anisometropia  (D) | Anisocoria  (mm) | Gaze deviation (PD) | | | | Media opacity |  |
| --- | --- | --- | --- | --- | --- | --- | --- | --- | --- | --- | --- |
|  |  |  |  |  |  | Vertical | Nasal | Temporal | Asymmetry |  | |
| 6-12 | ≥3.5 | ≥-2 | ≥2.25 | ≥1.5 | ≥1 | ≥8 | ≥5 | ≥8 | ≥8 | Any | |
| 12-36 | ≥3 | ≥-2 | ≥2 | ≥1 | ≥1 | ≥8 | ≥5 | ≥8 | ≥8 | Any | |
| 36-72 | ≥2.5 | ≥-1.25 | ≥1.75 | ≥1 | ≥1 | ≥8 | ≥5 | ≥8 | ≥8 | Any | |
| 72-240 | ≥2.5 | ≥-1 | ≥1.5 | ≥1 | ≥1 | ≥8 | ≥5 | ≥8 | ≥8 | Any | |
| >240 | ≥1.5 | ≥-0.75 | ≥1.5 | ≥1 | ≥1 | ≥8 | ≥5 | ≥8 | ≥8 | Any | |

**Supplementary Table 2.** Summary of all data

|  |  | **Gender** | **SER OD (Diopter)** | | **SER OS (Diopter)** | | **Prevalence (%)** | | | **Cut-off UCVA = 20/25** | | **Cut-off UCVA = 20/32** | | **Cut-off UCVA = 20/40** | |
| --- | --- | --- | --- | --- | --- | --- | --- | --- | --- | --- | --- | --- | --- | --- | --- |
| **Age** | **n** | **Male%** | **mean** | **se** | **mean** | **se** | **Myopia (SER≤-0.5D)** | **High Myopia (SER≤-6.0D)** | **Anisometropia (ΔSER >=1D)** | **sensitivity** | **Specificity** | **sensitivity** | **Specificity** | **sensitivity** | **Specificity** |
| 5 | 245 | 54.7% | 0.18 | 0.05 | 0.05 | 0.05 | 10.2% | 0.0% | 6.1% | 0.872 | 0.716 | 0.462 | 0.920 | 0.385 | 0.962 |
| 6 | 627 | 52.5% | 0.21 | 0.05 | 0.20 | 0.05 | 14.8% | 0.5% | 10.2% | 0.761 | 0.645 | 0.642 | 0.818 | 0.465 | 0.911 |
| 7 | 789 | 54.1% | -0.21 | 0.05 | -0.28 | 0.05 | 38.5% | 0.0% | 13.6% | 0.831 | 0.620 | 0.682 | 0.824 | 0.504 | 0.913 |
| 8 | 959 | 54.1% | -0.69 | 0.05 | -0.64 | 0.05 | 52.6% | 0.4% | 17.6% | 0.870 | 0.556 | 0.744 | 0.782 | 0.582 | 0.887 |
| 9 | 1233 | 52.4% | -1.13 | 0.05 | -1.11 | 0.05 | 67.2% | 0.6% | 21.4% | 0.892 | 0.681 | 0.767 | 0.826 | 0.597 | 0.900 |
| 10 | 1533 | 52.3% | -1.60 | 0.04 | -1.50 | 0.04 | 78.4% | 0.8% | 23.6% | 0.890 | 0.690 | 0.764 | 0.855 | 0.607 | 0.917 |
| 11 | 1699 | 50.4% | -1.98 | 0.04 | -1.78 | 0.04 | 85.8% | 1.5% | 26.2% | 0.903 | 0.702 | 0.796 | 0.828 | 0.652 | 0.903 |
| 12 | 1841 | 51.0% | -2.46 | 0.04 | -2.11 | 0.04 | 91.4% | 5.5% | 31.0% | 0.901 | 0.725 | 0.805 | 0.852 | 0.678 | 0.898 |
| 13 | 1805 | 48.6% | -2.94 | 0.04 | -2.76 | 0.04 | 93.5% | 4.4% | 23.7% | 0.943 | 0.720 | 0.879 | 0.860 | 0.775 | 0.907 |
| 14 | 1986 | 50.7% | -2.97 | 0.04 | -2.68 | 0.04 | 93.8% | 6.1% | 28.4% | 0.938 | 0.704 | 0.880 | 0.805 | 0.779 | 0.878 |
| 15 | 1569 | 54.3% | -3.24 | 0.05 | -2.88 | 0.05 | 95.0% | 8.8% | 28.5% | 0.934 | 0.741 | 0.881 | 0.826 | 0.790 | 0.895 |
| 16 | 265 | 58.5% | -2.97 | 0.12 | -2.72 | 0.13 | 94.7% | 6.4% | 25.7% | 0.932 | 0.726 | 0.825 | 0.871 | 0.746 | 0.919 |
| **ALL** | **14551** | **52.8%** | **-1.65** | **0.05** | **-1.52** | **0.05** | **78.3%** | **3.5%** | **24.1%** | **0.913** | **0.667** | **0.824** | **0.830** | **0.699** | **0.906** |

**Supplementary Table 3. P values for comparison of the SER between each age group (t.test for Figure 1B)**

| **Female (Age)** | 5 | 6 | 7 | 8 | 9 | 10 | 11 | 12 | 13 | 14 | 15 | 16 |
| --- | --- | --- | --- | --- | --- | --- | --- | --- | --- | --- | --- | --- |
| 5 | p = 1 | 0.07623 | 1.37E-08 | < 2.2e-16 | < 2.2e-16 | < 2.2e-16 | < 2.2e-16 | < 2.2e-16 | < 2.2e-16 | < 2.2e-16 | < 2.2e-16 | < 2.2e-16 |
| 6 |  | 1 | 6.74E-11 | < 2.2e-16 | < 2.2e-16 | < 2.2e-16 | < 2.2e-16 | < 2.2e-16 | < 2.2e-16 | < 2.2e-16 | < 2.2e-16 | < 2.2e-16 |
| 7 |  |  | 1 | 1.05E-07 | < 2.2e-16 | < 2.2e-16 | < 2.2e-16 | < 2.2e-16 | < 2.2e-16 | < 2.2e-16 | < 2.2e-16 | < 2.2e-16 |
| 8 |  |  |  | 1 | 1.01E-13 | < 2.2e-16 | < 2.2e-16 | < 2.2e-16 | < 2.2e-16 | < 2.2e-16 | < 2.2e-16 | < 2.2e-16 |
| 9 |  |  |  |  | 1 | 1.82E-10 | < 2.2e-16 | < 2.2e-16 | < 2.2e-16 | < 2.2e-16 | < 2.2e-16 | < 2.2e-16 |
| 10 |  |  |  |  |  | 1 | 2.51E-07 | < 2.2e-16 | < 2.2e-16 | < 2.2e-16 | < 2.2e-16 | < 2.2e-16 |
| 11 |  |  |  |  |  |  | 1 | 2.37E-16 | < 2.2e-16 | < 2.2e-16 | < 2.2e-16 | < 2.2e-16 |
| 12 |  |  |  |  |  |  |  | 1 | 8.98E-10 | 6.2E-11 | < 2.2e-16 | 0.01982 |
| 13 |  |  |  |  |  |  |  |  | 1 | 0.3819 | 3.14E-06 | 0.6094 |
| 14 |  |  |  |  |  |  |  |  |  | 1 | 0.000221 | 0.3792 |
| 15 |  |  |  |  |  |  |  |  |  |  | 1 | 0.007061 |
| 16 |  |  |  |  |  |  |  |  |  |  |  | 1 |
|  |  |  |  |  |  |  |  |  |  |  |  |  |
| **Male (Age)** | 5 | 6 | 7 | 8 | 9 | 10 | 11 | 12 | 13 | 14 | 15 | 16 |
| 5 | p = 1 | 0.4254 | 7.55E-07 | < 2.2e-16 | < 2.2e-16 | < 2.2e-16 | < 2.2e-16 | < 2.2e-16 | < 2.2e-16 | < 2.2e-16 | < 2.2e-16 | < 2.2e-16 |
| 6 |  | 1 | 8.16E-10 | < 2.2e-16 | < 2.2e-16 | < 2.2e-16 | < 2.2e-16 | < 2.2e-16 | < 2.2e-16 | < 2.2e-16 | < 2.2e-16 | < 2.2e-16 |
| 7 |  |  | 1 | 6.29E-09 | < 2.2e-16 | < 2.2e-16 | < 2.2e-16 | < 2.2e-16 | < 2.2e-16 | < 2.2e-16 | < 2.2e-16 | < 2.2e-16 |
| 8 |  |  |  | 1 | 6.77E-07 | < 2.2e-16 | < 2.2e-16 | < 2.2e-16 | < 2.2e-16 | < 2.2e-16 | < 2.2e-16 | < 2.2e-16 |
| 9 |  |  |  |  | 1 | 1.44E-10 | < 2.2e-16 | < 2.2e-16 | < 2.2e-16 | < 2.2e-16 | < 2.2e-16 | < 2.2e-16 |
| 10 |  |  |  |  |  | 1 | 5.69E-08 | < 2.2e-16 | < 2.2e-16 | < 2.2e-16 | < 2.2e-16 | < 2.2e-16 |
| 11 |  |  |  |  |  |  | 1 | 2.79E-06 | < 2.2e-16 | < 2.2e-16 | < 2.2e-16 | < 2.2e-16 |
| 12 |  |  |  |  |  |  |  | 1 | < 2.2e-16 | < 2.2e-16 | < 2.2e-16 | < 2.2e-16 |
| 13 |  |  |  |  |  |  |  |  | 1 | 0.1311 | 0.04067 | 0.6752 |
| 14 |  |  |  |  |  |  |  |  |  | 1 | 0.000332 | 0.6784 |
| 15 |  |  |  |  |  |  |  |  |  |  | 1 | 0.1119 |
| 16 |  |  |  |  |  |  |  |  |  |  |  | 1 |

**Supplementary Table 4. Age dependent mean and 95% CI of refractive error for children with different level of UCVA.**

| UCVA  Age | 20/16 | 20/20 | 20/25 | 20/32 | 20/40 | 20/50 | 20/63 | 20/100 | 20/200 |
| --- | --- | --- | --- | --- | --- | --- | --- | --- | --- |
| 5 | N/A | 0.22 (0.17~0.27)  n=193 | 0.16 (0.08~0.23)  n=135 | 0.1 (-0.05~0.25)  n=108 | 0.15 (-0.65~0.95)  n=22 | -0.5 (-1.5~0.5)  n=16 | -1.19 (-2.04~-0.35)  n=9 | -1.75 (-2.99~-0.51)  n=3 | 0.69 (0.09~1.28)  n=4 |
| 6 | 0.27 (0.13~0.4)  n=33 | 0.25 (0.21~0.3)  n=383 | 0.21 (0.14~0.29)  n=328 | 0.45 (0.29~0.6)  n=209 | 0.23 (0.04~0.43)  n=129 | 0.18 (-0.27~0.63)  n=72 | -0.54 (-1.17~0.08)  n=53 | -0.78 (-1.72~0.16)  n=23 | -0.47 (-2.56~1.62)  n=24 |
| 7 | 0.19 (0.04~0.34)  n=57 | 0.17 (0.1~0.25)  n=296 | 0.09 (0~0.18)  n=390 | 0.06 (-0.07~0.2)  n=293 | -0.27 (-0.48~-0.06)  n=187 | -0.75 (-1.02~-0.48)  n=139 | -1.33 (-1.62~-1.04)  n=111 | -1.7 (-2.27~-1.14)  n=71 | -2.52 (-3.39~-1.66)  n=34 |
| 8 | 0.16 (0.06~0.26)  n=83 | 0.02 (-0.06~0.09)  n=280 | -0.11 (-0.2~-0.01)  n=315 | 0.07 (-0.09~0.22)  n=342 | -0.56 (-0.75~-0.36)  n=253 | -1.15 (-1.36~-0.95)  n=230 | -1.48 (-1.79~-1.17)  n=167 | -2.5 (-2.76~-2.24)  n=190 | -2.56 (-3.21~-1.92)  n=58 |
| 9 | 0.06 (-0.02~0.14)  n=109 | 0.01 (-0.07~0.08)  n=345 | -0.12 (-0.21~-0.02)  n=368 | -0.59 (-0.73~-0.44)  n=327 | -1.12 (-1.27~-0.96)  n=327 | -1.51 (-1.68~-1.35)  n=326 | -2.03 (-2.22~-1.84)  n=290 | -2.64 (-2.88~-2.4)  n=272 | -3.69 (-4.14~-3.24)  n=102 |
| 10 | -0.09 (-0.18~0.01)  n=95 | -0.1 (-0.18~-0.02)  n=342 | -0.33 (-0.42~-0.24)  n=409 | -0.86 (-0.97~-0.74)  n=420 | -1.44 (-1.58~-1.3)  n=399 | -1.75 (-1.89~-1.62)  n=419 | -2.46 (-2.59~-2.32)  n=469 | -3.18 (-3.34~-3.02)  n=367 | -4.03 (-4.32~-3.73)  n=146 |
| 11 | 0.03 (-0.08~0.15)  n=92 | -0.2 (-0.28~-0.11)  n=332 | -0.47 (-0.58~-0.36)  n=354 | -0.99 (-1.12~-0.87)  n=377 | -1.63 (-1.76~-1.5)  n=438 | -2.03 (-2.16~-1.9)  n=501 | -2.7 (-2.82~-2.58)  n=559 | -3.18 (-3.32~-3.03)  n=520 | -4.02 (-4.25~-3.79)  n=225 |
| 12 | -0.1 (-0.36~0.16)  n=22 | -0.22 (-0.3~-0.15)  n=426 | -0.73 (-0.84~-0.62)  n=295 | -1.19 (-1.34~-1.05)  n=374 | -1.79 (-1.91~-1.68)  n=418 | -2.33 (-2.45~-2.2)  n=500 | -2.93 (-3.04~-2.81)  n=710 | -3.58 (-3.71~-3.45)  n=601 | -4.53 (-4.74~-4.33)  n=336 |
| 13 | -0.06 (-0.25~0.12)  n=31 | -0.26 (-0.36~-0.16)  n=220 | -0.74 (-0.88~-0.59)  n=211 | -1.34 (-1.49~-1.18)  n=261 | -2.18 (-2.31~-2.05)  n=354 | -2.73 (-2.85~-2.61)  n=508 | -3.33 (-3.42~-3.24)  n=830 | -3.78 (-3.88~-3.69)  n=762 | -4.43 (-4.58~-4.28)  n=433 |
| 14 | -0.29 (-0.57~-0.01)  n=20 | -0.31 (-0.4~-0.22)  n=317 | -0.61 (-0.76~-0.46)  n=216 | -1.34 (-1.5~-1.19)  n=250 | -2.01 (-2.17~-1.84)  n=388 | -2.58 (-2.73~-2.44)  n=513 | -3.23 (-3.34~-3.12)  n=853 | -3.75 (-3.85~-3.66)  n=813 | -4.54 (-4.66~-4.42)  n=602 |
| 15 | -0.14 (-0.75~0.47)  n=7 | -0.35 (-0.44~-0.26)  n=244 | -0.76 (-0.93~-0.6)  n=163 | -1.35 (-1.57~-1.14)  n=176 | -2.07 (-2.26~-1.87)  n=278 | -2.65 (-2.79~-2.5)  n=368 | -3.34 (-3.47~-3.21)  n=650 | -4 (-4.12~-3.88)  n=673 | -4.74 (-4.86~-4.62)  n=579 |
| 16 | -0.5 (-1.12~0.12)  n=3 | -0.26 (-0.47~-0.04)  n=45 | -1.24 (-1.57~-0.92)  n=29 | -1.36 (-1.69~-1.03)  n=59 | -1.64 (-2.08~-1.21)  n=40 | -2.4 (-2.67~-2.14)  n=71 | -3.44 (-3.74~-3.13)  n=105 | -4.05 (-4.3~-3.8)  n=102 | -4.45 (-4.79~-4.1)  n=76 |

**Data are presented as: Mean (95%CI) Number of cases. CI: confidence interval. UCVA: Uncorrected Visual Acuity.**

**Supplementary Table 5. P values for comparison of the Anisometropia between each age group (t.test for Figure 6)**

| **Female (Age)** | 5 | 6 | 7 | 8 | 9 | 10 | 11 | 12 | 13 | 14 | 15 | 16 |
| --- | --- | --- | --- | --- | --- | --- | --- | --- | --- | --- | --- | --- |
| 5 | p = 1 | 0.8682 | 0.1161 | 0.8243 | 0.9856 | 0.08591 | 0.001726 | 3.05E-09 | 0.0027 | 3.11E-06 | 3.12E-08 | 0.009526 |
| 6 |  | 1 | 0.1326 | 0.9537 | 0.8383 | 0.03982 | 0.000309 | 2.1E-11 | 0.000437 | 9.28E-08 | 4.22E-10 | 0.006206 |
| 7 |  |  | 1 | 0.1431 | 0.07462 | 0.000147 | 6.4E-08 | < 2.2e-16 | 4.6E-08 | 4.32E-13 | 9.05E-16 | 0.000424 |
| 8 |  |  |  | 1 | 0.7892 | 0.03141 | 0.00019 | 5.86E-12 | 0.000258 | 3.6E-08 | 1.39E-10 | 0.005496 |
| 9 |  |  |  |  | 1 | 0.05274 | 0.000347 | 7.27E-12 | 0.000471 | 5.55E-08 | 1.94E-10 | 0.007746 |
| 10 |  |  |  |  |  | 1 | 0.07913 | 8.81E-08 | 0.1303 | 0.000199 | 1.36E-06 | 0.07386 |
| 11 |  |  |  |  |  |  | 1 | 0.000211 | 0.6729 | 0.05413 | 0.001434 | 0.3124 |
| 12 |  |  |  |  |  |  |  | 1 | 8.84E-06 | 0.04665 | 0.627 | 0.4799 |
| 13 |  |  |  |  |  |  |  |  | 1 | 0.009968 | 0.000107 | 0.2299 |
| 14 |  |  |  |  |  |  |  |  |  | 1 | 0.1442 | 0.8539 |
| 15 |  |  |  |  |  |  |  |  |  |  | 1 | 0.6363 |
| 16 |  |  |  |  |  |  |  |  |  |  |  | 1 |
|  |  |  |  |  |  |  |  |  |  |  |  |  |
| **Male (Age)** | 5 | 6 | 7 | 8 | 9 | 10 | 11 | 12 | 13 | 14 | 15 | 16 |
| 5 | p = 1 | 0.06182 | 0.1706 | 0.000258 | 0.01543 | 0.000195 | 2.56E-07 | 8.94E-12 | 5.62E-07 | 6.83E-11 | 9.2E-14 | 0.000193 |
| 6 |  | 1 | 0.5478 | 0.04619 | 0.5193 | 0.06218 | 0.000349 | 3.67E-08 | 0.00075 | 2.48E-07 | 4.21E-10 | 0.007994 |
| 7 |  |  | 1 | 0.008326 | 0.2114 | 0.008538 | 1.43E-05 | 2.45E-10 | 3.17E-05 | 2.4E-09 | 1.29E-12 | 0.002456 |
| 8 |  |  |  | 1 | 0.1867 | 0.6646 | 0.1641 | 0.000945 | 0.2662 | 0.002985 | 5.28E-05 | 0.1578 |
| 9 |  |  |  |  | 1 | 0.2779 | 0.004931 | 2.43E-06 | 0.009849 | 1.22E-05 | 5.14E-08 | 0.02512 |
| 10 |  |  |  |  |  | 1 | 0.0342 | 1.33E-05 | 0.06723 | 7.65E-05 | 1.84E-07 | 0.08284 |
| 11 |  |  |  |  |  |  | 1 | 0.03611 | 0.7202 | 0.08345 | 0.003724 | 0.5414 |
| 12 |  |  |  |  |  |  |  | 1 | 0.0113 | 0.7218 | 0.422 | 0.5694 |
| 13 |  |  |  |  |  |  |  |  | 1 | 0.03134 | 0.000757 | 0.4168 |
| 14 |  |  |  |  |  |  |  |  |  | 1 | 0.2476 | 0.7142 |
| 15 |  |  |  |  |  |  |  |  |  |  | 1 | 0.3059 |
| 16 |  |  |  |  |  |  |  |  |  |  |  | 1 |
